# Atlanta Academy of Medicine

**Published:** 1875-02

**Authors:** 


### Atlanta Academy of Medicine.

A. W. CALHOUN, M.D., REPORTER.

ATLANTA, December 15, 1874.

Dr. Armstrong, President, presiding.

Dr. W. F. Westmoreland continued the report of a case of stricture, twenty-two days after the perineal section; the operation for the relief of the stricture was attempted by bougies, etc. There was complete occlusion, and no kind of bougie could be introduced. Upon trying the urethratome, a portion of the instrument was broken off in the urethra; but the attempt was continued until Thompson's divulsor was entered, when the stricture was broken up and the broken portion of instrument was brought out between the blades of the divulsor. Patient is now doing well, and bougies are being regularly introduced. Adjourned.

ATLANTA, GA., December 23, 1874.

Dr. Armstrong, President, presiding.

Dr. Taliaferro reported the case of a man who was much reduced in consequence of a chronic diarrhœa, lasting six months, and who had had convulsions, and when seen by him was in a comatose condition. On the night previous he had been to a wedding, and had eaten heartily. Upon administering chloroform the convulsions were checked. Also, thirty grains ipecac, mixed in a very little water was given, and in three hours a second dose of the same size. Upon seeing him on the following morning, he learned he had vomited particles of meat, and found him partially paralyzed, the right arm completely. He was unable to articulate, and it seemed he must die. One-half grain of opium and 20 grains ipecac were given every three or four hours until ℨj. of the latter had been taken. He rested well during the night, and was comfortable next morning. The partial paralysis was gone, and he was again able to articulate. The remedies were repeated, and he continued to improve. Castor oil was freely and frequently given, without immediate avail, but finally acted, and he is now entirely conscious and doing well.

Dr. J. M. Johnson thought it was difficult to always get a good article of ipecac. The root made into tea will vomit, but the powdered article that is purchased is very often wholly inert. It has the proper taste and odor, but the effect is not good. If you powder the root and make a decoction it will almost invariably produce vomiting. Twenty grains of pure ipecac must necessarily vomit; one grain often will. Where it does not vomit he would suppose the ipecac to be impure. In Dr. T.'s case he thinks the reflex action caused the convulsions, but not the paralysis. If effusion took place it must have been absorbed.

Dr. Battey—The genuineness of ipecac root ought not to be doubtful. The root is peculiar; its bark is, relatively, exceedingly thick, whilst the inner, ligneous portion is but a small, very tough cord, which resists greatly the action of the powdering mill. The cortical layer is starchy and fragile, powders easily, and readily breaks up into annular fragments arranged upon the ligneous cord like beads upon a string. The usual high price of ipecac which has obtained for years past opens very strong inducement for adulteration in the hands of the unscrupulous. In reference to Dr. T.'s case, thinks it not necessary to have any *lesion* of the brain to produce paralysis. He can imagine troubles of digestion being reflected and bringing about such a state of things as in this case; thinks he himself has had some such cases. He narrated cases of two men eating, late at night, certain articles, when one died from cerebritis and the other was very sick from disturbance of the brain. If digestive troubles produce coma, and recovery follows, why should it not paralysis, with subsequent recovery?

Dr. Boring had a patient who ate raw turnips, and had colic in consequence. He took whisky, and afterwards had convulsions, followed by paralysis of the right side, and is still paralyzed, (now five weeks since the convulsions.) Thinks the indigestible turnips produced the paralysis. Another case in a little girl, who, from eating cocoanut candy, had convulsions, paralysis, etc. He gave an enema, and she passed pieces of the cocoanut; whereupon the paralysis passed away. If a child is thus affected, why not an adult?

Dr. Holmes said that ingesta can produce convulsions and coma, as the effect of reflex action. Paralysis, as a rule, is the result of a lesion. When there is any renal trouble, chronic diarrhœa and gastric irritation frequently result from retained urea in the blood, it being vicariously eliminated in that way, and, failing to be eliminated, can end in convulsions, coma, etc.

Dr. Armstrong reported a case of a man who, three weeks before, had fallen fifteen feet from a bridge, and fractured the metatarsal bone of the little toe of the left foot; also received an injury in the side. He had cramps in the left limb, and in a few hours found he could not pass his urine, when it became necessary to use the catheter. There is now atony of the bladder, and it has to be emptied by means of the catheter two or three times daily. Strychnine and quinine have been given, but there is no improvement in the bladder, which now is the main source of trouble, the other injuries improving rapidly.

Dr. Woodhull remarked that the difficulty might be a shock to the sympathetic nervous system, and thought ergot might have a good effect in stimulating the muscular coat of the bladder.

Dr. J. M. Johnson thought time would cure the case, and was of the opinion that concussion of the spine had produced paralysis of the motor fibres supplying the bladder. Recovering from the effects of the concussion, he would recover from the paralysis.

Dr. Calhoun reported a case upon which he had made the operation known as peritomy. The patient was a girl, aged sixteen years, who, when three years old, had purulent ophthalmia in both eyes, which left the lids covered with granulations (granular ophthalmia), and the cornea of each eye covered with a thick deposit, filled with innumerable blood vessels, constituting complete pannus. The eyes remained in this chronically inflamed condition till she came under treatment, several months ago, and had so little vision (being unable even to count fingers when held directly before her) that she had to be led wherever she went. By daily applications of nitrate of silver and sulphate of copper to the mucous lining of the lids for several months, the granulations were ultimately completely removed, and also the pannus from the left cornea, thus affording her excellent vision in this eye. But notwithstanding the disappearance of the granulations from the right eye, the pannus upon the cornea still continued, leaving the eye almost as blind as before the treatment was begun. The following operation was made to relieve the cornea of the opacity and vascularity. Seizing, by means of forceps, a portion of the conjunctiva, so as to steady the eye, with a pair of curved scissors a circular incision was made through the conjunctiva, entirely around the cornea, and parallel to and about one and a half lines back from its edge. This circular portion of conjunctiva was then dissected off close up to the edge of the cornea, cutting away at the same time the sub-conjunctival tissue covering the corresponding portion of the sclerotic coat. Thus the sclerotic was laid bare for a space of one and a half lines in width, and extending entirely around and up to the edge of the cornea. By this means the blood vessels, running from the conjunctiva into the cornea, were totally cut off. The patient made a rapid recovery, with but little inflammation resulting from the operation, and a firm white cicatrix occupies the part from which the conjunctiva and sub-conjunctival tissues were removed. The opacity of the cornea gradually and completely cleared up, and now the patient, without any further treatment than occasionally touching the lid with sulphate of copper, has perfectly recovered, with normal vision in this as well as in the other eye.

Dr. J. M. Johnson had seen many such cases as the one just reported, and observed that every remedy had failed to afford relief, that is, the restoration of vision. He saw this case before the operation, and saw her operated upon, and has seen her since her recovery. He is satisfied as to the success of the operation in removing the pannus and restoring the girl to excellent sight.

Dr. Armstrong had seen two such operations in the Royal Ophthalmic Hospital of London, both resulting successfully. In these cases there was some vision, but a cloudiness covered the cornea. In his own practice he had had a case in which the vascularity was relieved, but the cloudiness of the cornea still remained after the operation. In another similar case, he was more successful in removing the corneal opacity.

---

ATLANTA, GA., December 29, 1874.

The President, Dr. W. S. Armstrong, presiding.

Dr. Boring continued the report of his case of supposed gout. She was still in bed, and no improvement had taken place.

Dr. Taliaferro had examined the urine for uric acid, but found none. She was afterwards put upon citrate of lithia, to eliminate the uric acid from the blood. He is now in doubt about its being gout, and in view of its being a nervous trouble, suggests that she be put upon 1-12 gr. phosphorus, three times daily, and cod liver oil, and that she wean her child.

Dr. W. F. Westmoreland thinks it was a case of neurosis. He cannot call anything gout or rheumatism without inflammation, or some of the rational signs of the disease.

Dr. Armstrong continued the report of his case of atomy of the bladder, produced by a fall. There was but little improvement. By a great effort he passes a few drops of water. In general, the patient is doing well.

Dr. W. F. Westmoreland reports a case of injury, simply to show the tenacity of life. Was called in consultation to see a man who had been pressed between two cars, passing each other. He was rational when seen. Both collar bones were broken, also the sternum and six or eight ribs, which were driven into the lung. The heart was displaced, and one lung lacerated, and yet he lived eight or ten hours. He would not have believed that a man could live one moment after such an injury.

Dr. Battey mentioned the case of a man, a mill sawyer, who was run over by twenty or thirty logs, pressing him together, and producing innumerable fractures, ~~and~~ constituting him a surgical curiosity. He recovered completely, and afterwards became a soothsayer in his region, as a consequence of his miraculous escape.

Dr. W. F. Westmoreland asked the members of the Academy if they had recently met with any erysipelas in the city? Some operations he had made had taken on an erysipelatous inflammation, and resulted badly.

Drs. Baird, Taliaferro, Boring, Battey, Armstrong, and J. M. Johnson had seen cases.

Dr. Battey inquired as to what treatment the members bring to bear upon erysipelas? In idiopathic erysipelas his local treatment had not been satisfactory. Tinc. iodine, nit. silver, etc., in his hands had done no service. The most serviceable local treatment is the soothing poultice.

Dr. Boring applied tinct. iodine, and used cold cranberry poultices, and gave internally, every four hours, twenty drops of a solution of 60 gr. quinine, to ℥j mur. tinct. iron. His patient recovered rapidly.

Dr. W. F. Westmoreland thought local treatment had nothing to do with curing this disease. It does no more than relieve the patient. It is a constitutional disease, and this must be cured. Traumatic and idiopathic erysipelas are alike constitutional diseases. It does not always occur in a wound, but sometimes elsewhere. It is the constitutional remedy that does the work. If it is palegmonous, punctures are useful. It is the constitutional treatment that cures. Thinks the idea of the disease being contagious, and of isolating individuals thus affected, a foolish one; the cause is atmospheric.

Dr. J. M. Johhson said that in 1847 or '48 he read an article from Dr. Dickson (then of Charleston) upon erysipelas, in which he suggested tincture iron, quinine and calomel as the treatment. Dr. D. considered it a malarial poison. Three years afterward he encountered a severe epidemic, in which mortification and death would ensue in a short time. In this epidemic he tested these remedies, pushing them as far as the patient could bear. He has not found much benefit from local treatment. ℨj. calomel to a tablespoonful of lard, as a covering to exclude the air, is a good application. In this same epidemic he used nit. silver to prevent the spread of the disease, but no benefit was derived. We must treat the disease constitutionally and by nourishing with stimulants and nourishing food. As an application to the parts, two parts of castor oil and one part of collodion was recommended by Dr. Twitty, of Mitchell county, as having been used with immense benefit. It is soothing and better than a poultice.

Dr. Battey thinks there is a lamentable need of knowlege as to the natural history of erysipelas and other diseases. In Parisian hospitals, the beet sugar water treatment seemed to do as much good as in others where the disease was more vigorously treated.

Dr. J. M. Johnson—What injury could result from quinine and iron? If the bowels are inactive, what is better than calomel? There is a poison generated by just such weather as we are now having, which produces this disease. Magendie used beet root sugar and water, and lost much of his practice. His confreres used iron and quinine, and had their hospitals full. There must have been some cause for this. He is not one of those who disbelieve in medicines. The "Vis medicatrix naturæ" will not do for this age. Calomel and opium are indispensable remedies, and do good where beet root sugar would have been of no service.

Adjourned.

---

Atlanta, Ga., January 5, 1875.

Dr. Armstrong, President, presiding.

Dr. W. F. Westmoreland requested Dr. Boring to report the progress of the case of erysipelas in the man upon whom the operation for stricture and perineal section was made.

Dr. Boring reported that the swelling of the scrotum and penis had greatly subsided. Every four hours five grains each of calomel and dover's powder had been given him; also, every four hours, forty drops of a solution of quinine in tinct. iron (ʒj. to ℥j.) He is doing well. Thinks this epidemic influence is the cause of the disease in this case.

Dr. Baird asks why calomel is given in this disease?

Dr. W. F. Westmoreland gives it partially empirically, and with the view of checking the constitutional disease. Does not give it as a specific. In some epidemics he has seen other purgatives (as saline) given with the same effect as calomel. Cannot say how it affects the disease.

Dr. Baird inquired if it had been found specially beneficial in erysipelas?

Dr. W. F. Westmoreland, in reply, mentioned a case where a dose of calomel had always checked repeated recurrences of the disease.

Dr. Miller mentioned that, in erysipelas, he had uniformly used cathartics and twenty to thirty grains iodide potassium in each dose, given frequently during the day, and mostly with good effect. In reply to the incidental remark of Dr. Baird, that he considered mercury a specific in syphilis, Dr. Miller remarked that he did not so consider it; in fact, thought it unnecessary to give mercury until some symptoms of the secondary stage had made their appearance. It is a well known fact that syphilis, in its first stages, often gets well without any treatment, and why should a patient be tortured by a mercurial course of treatment, when, in all probability, he would have recovered without it? Moreover, how could we know that the treatment had accomplished anything, if no secondary symptoms had appeared? We might as well have supposed that the patient would have gotten well without the mercury. The experience and experiments of large hospitals prove this fact.

Dr. J. G. Westmoreland was of the opinion that, if Hunterian chancre exists, we may safely look for secondary symptoms.

Dr. Calhoun replied to the inquiry, as to what the experiments in European hospitals proved in reference to the mercurial and non-mercurial treatment of syphilis, that he had witnessed in the hospitals of Berlin and Vienna a series of experiments, showing the curability of the disease in its first stages just as well by non-constitutional or non-mercurial treatment as by any regular mercurial course. The only difference (if any) was, that the patients treated wholly by mercury were cured more rapidly; but their cures were not more perfect than the others. No attention was given to the fact that one chancre was soft and another hard, but all classed under the general term of chancre, some being treated, others not, except in so far as cleanliness was concerned, and all recovering alike. It is the opinion of Hebra, in Vienna, that the secondary and tertiary stages of syphilis are incurable—that an individual thus once affected, though apparently cured, will, if he live long enough, have the disease re-appearing in some of its various types. To show this, a case was on one occasion demonstrated in whom, thirty or forty years after the first appearance of the disease and its supposed cure, the syphilis was again well marked, although there had been no cause for fresh contagion. It is also his opinion that several children of a syphilitic father may escape, and the disease still show itself in the third or fourth child.

Dr. W. F. Westmoreland said that, from the remarks of gentlemen, it would appear that they were impressed with the idea that there were two varieties of syphilis, when the truth is there is only one; that it was evident from remarks to-night that members were disposed to make chancroid a variety of syphilis, when the truth is there is as much difference between chancroid and syphilis as any two diseases in the nomenclature of medicine. One (chancroid) is an entirely local affection, while the other (syphilis) is purely a constitutional trouble from the commencement—that, at or before the local development or *Hunterian* or *infectant* chancre, as they were formerly called, make their appearance, the system has been impressed, as has been so often demonstrated in other localities. Syphilis, then, is a constitutional trouble from its inception, and is alone amenable to constitutional remedies, while chancroid, *soft*, *non-indurated* or non-infectant chancre of the older authors, is as truly a local affection, and is alone amenable to local remedies. That whatever may be the mode of treatment or extent of its ravages, it can never become constitutional, or produce syphilitic symptoms, no more than a sore of any other character in any other locality.

Anti-syphilitic remedies in chancroid will not accomplish any good, but are often sources of great evil, and should not be thought of, while in syphilis we rely entirely and with great certainty upon constitutional remedies. In cases where, from previous treatment or other causes, we cannot with certainty say whether we have syphilis or chancroid, for many reasons, which I don't propose to discuss to-night, it is best to withhold all syphilitic remedies until we have unmistakable evidence of syphilis. He contended that the old idea, upon which so many acted, that by anti-syphilitic remedies we can prevent the system becoming involved, was exploded when it was proven that the constitution is impressed before the first sore makes its appearance. He said, as to the idea of leaving it to nature, or soap and water, he felt confident he could never be a convert; that whilst perhaps all cases of chancroid would sooner or later recover without the use of active curative remedies, still, all who have watched with care the rapidly curative effects of appropriate remedies would not be disposed, he felt confident, to leave the sores without treatment; that, in syphilis, he would certainly insist upon appropriate remedies, and should feel himself highly censurable were he to withhold remedies that so constantly act so happily. This will perhaps do for hospitals, where experiments are so rife, but you will find that the conductors of like experiments do not act upon their results in private practice.

As to the possibility of eradicating syphilis from the system, he said, after hearing Ricord's lectures, he believed that we could not; but, at present, was firmly convinced we could. He mentioned several cases that had come under his observation. He contended that the greatest trouble was to say when it was eradicated. In many cases, of well-established syphilis, believes it to be entirely eradicated.

---

ROBERT BATTEY, M.D., REPORTER.

---

ATLANTA, GA., Jan. 12, 1875.

Dr. John M. Johnson, President, presiding.

Dr. John M. Johnson read an essay upon dysmenorrhœa.

Dr. Taliaferro remarked that dysmenorrhœa is not a disease, but only a symptom of disease, and arises from a variety of causes, and is attendant upon a large variety of pathological conditions. The corset, or undue tightness of the clothing, even, induce uterine and ovarian hyperæmia, and consequent dysmenorrhœa. Indolence and luxury often produce the symptom dysmenorrhœa. Thinks the treatment recommended in the essay admirable when viewed as a paliative course, but impotent for the cure of the pathological states which produce this symptom.

Dr. W. F. Westmoreland remarked that he agreed with the essayist in estimating the great importance of the views advanced for the prevention of the diseased conditions which induce dysmenorrhœa, by proper attention to early hygiene and development. Thinks Dr. J. is sustained, in many instances, in viewing dysmenorrhœa as a disease of function at first, afterwards to be followed by organic lesions as a consequence and not as a cause.

Dr. Woodhull mentioned a case where dysmenorrhœa had been cured, as it were, by accident, having been relieved by the exchange of an ill-fitting corset for one of more rational construction.

Dr. John M. Johnson remarked upon the great difference in treatment of dysmenorrhœa of forty years ago as compared with the present. He has tried the old method and the new; found the old way by far the most effective. Does not regard dysmenorrhœa as a disease, but only a diseased function. Does not believe that there is any connection whatever between ovulation and menstruation. Believes that menstruation is merely a provision for irrigation and softening and preparation of the uterus for the subsequent function of gestation. Simple dysmenorrhœa, when the paroxysm passes over, ordinarily leaves no symptoms indicative of organic disease. Thinks we ought to treat the symptom, persevere in treating and relieving the functional disturbances, until the normal habit is established permanently. Thinks obesity in man a great practical error in homoculture, impairing vigor and usefulness in the world; this ought to be corrected by proper dietetic discipline in early life; eat lean meat; avoid fats and bread. Raise children on the principles which obtain in the training of pugilists. Adjourned.

---

ATLANTA, GA., Jan. 19, 1875.

Dr. V. H. Taliaferro, Vice-President, presiding.

Dr. Armstrong continued report of case of atomy of the bladder. The patient is nearly recovered; the function of the bladder is regained. There is still great tenderness of the lower dorsal vertebræ and short ribs of the left side.

Dr. DeWitt corrected report of his treatment of powder stains. He used mask of tarlton, and this covered with thin slices of light-bread, and not bread poultice, as reported. The whole is then covered with tarlton, and water is continuously dropped upon the dressing to irrigate and wash out the grains of powder.

Dr. Boring reported case of epistaxis, treated by cording the left arm, and afterwards the right arm. On removing the cord from the left arm the bleeding recommenced, but soon subsided entirely. He had previously relieved epistaxis in five minutes by the same method. Does not explain the *modus operandi;* only knows the result.

Dr. Armstrong has been accustomed to rely upon posterior and anterior plugging of the nasal passage.

Dr. Battey suggested the digital compression of the artery in the upper lip, as a modern and rational treatment.

Dr. Woodhull suggested the elevation of the arms above the head.

Dr. Palmer suggested that a quack had used the cording of the extremities successfully for uterine hemorrhage.

Dr. Baird thought the arrest of bleeding results from cutting off a portion of blood from the general circulation.

Dr. Battey thought mental impression might be so strong as to arrest epistaxis. Adjourned.

